# Weil’s Disease

**Published:** 1888-06

**Authors:** 


### WEIL'S DISEASE.

Within the last two years, another disease, bearing a man's name, has been described and discussed. We have now Bright's, Addison's, Grave's or Basedow's, Raynaud's Friedreich's, Thomsen's, and Menière's diseases, besides Argyll-Robertson's pupils, Colles's fracture, and several surgeons' amputations. There are numerous objections to the practice of employing men's names to distinguish diseases. The taunt of the Psalmist against the heathen calling their lands after their own names does not apply to the present case, as the names in question, surnames of distingushed observers, are invariably attached by others to the diseases which these observers first described without the aid and convenience of nomenclature. Nevertheless, the practice is objectionable; it implies the glorification of men through disease, and, as part of a system of nomenclature, is faulty. Weil's disease is so-called for the usual reason—that is to say, it was first described by a physician of that name. About thirty cases have been noted, including those first detailed by Dr. Weil only two years ago. Dr. Fiedler has since written on the new disorder in the *Deutsch. Archiv,* Vol. XLII, part 4, page 281. His paper has already been ably condensed in the May number of the *American Journal of the Medical Sciences.* Fiedler concludes that the disease, first described by Weil in 1886, is not an abortive form of typhoid fever, as Dr. Weil has suggested. It is a distinct, acute infectious or toxic affection. The disease begins suddenly, without prodromal symptoms, but often with a chill. The constant symptoms are fever, headache, gastric disturbance, jaundice, and muscular pain, especially in the calves. The fever has a typical course, and lasts eight or ten days. Relapses have been observed. The spleen and liver are generally but not always swollen; the liver often becomes tender on pressure. Nephritis is often observed; herpes and erythema occur at times. The prognosis is generally favorable. Weil's disease is generally seen in hot weather, and men in the prime of life are the most subject to it. The cause is quite unknown, but butchers appear most liable to the disease, judging from the scanty statistics already at the disposal of the physicians who have studied Weil's disease.—*Exchange.*

